# Leaf‐IT: An Android application for measuring leaf area

**DOI:** 10.1002/ece3.3485

**Published:** 2017-10-18

**Authors:** Julian Schrader, Giso Pillar, Holger Kreft

**Affiliations:** ^1^ Department of Biodiversity, Macroecology and Biogeography Faculty for Forestry and Forest Ecology University of Goettingen Göttingen Germany

**Keywords:** App, functional ecology, functional trait, leaf area, logical agent, smartphone

## Abstract

The use of plant functional traits has become increasingly popular in ecological studies because plant functional traits help to understand key ecological processes in plant species and communities. This also includes changes in diversity, inter‐ and intraspecific interactions, and relationships of species at different spatiotemporal scales. Leaf traits are among the most important traits as they describe key dimensions of a plant's life history strategy. Further, leaf area is a key parameter with relevance for other traits such as specific leaf area, which in turn correlates with leaf chemical composition, photosynthetic rate, leaf longevity, and carbon investment. Measuring leaf area usually involves the use of scanners and commercial software and can be difficult under field conditions. We present Leaf‐IT, a new smartphone application for measuring leaf area and other trait‐related areas. Leaf‐IT is free, designed for scientific purposes, and runs on Android 4 or higher. We tested the precision and accuracy using objects with standardized area and compared the area measurements of real leaves with the well‐established, commercial software WinFOLIA using the Altman–Bland method. Area measurements of standardized objects show that Leaf‐IT measures area with high accuracy and precision. Area measurements with Leaf‐IT of real leaves are comparable to those of WinFOLIA. Leaf‐IT is an easy‐to‐use application running on a wide range of smartphones. That increases the portability and use of Leaf‐IT and makes it possible to measure leaf area under field conditions typical for remote locations. Its high accuracy and precision are similar to WinFOLIA. Currently, its main limitation is margin detection of damaged leaves or complex leaf morphologies.

## 1 INTRODUCTION

Plant functional traits describe ecologically relevant morphological, anatomical, biochemical, physiological, or phenological features of individuals and species and provide information about the environmental constraints a plant faces (Pérez‐Harguindeguy et al., 2013). The study of functional traits allows, among others, to compare habitats with little taxonomic overlap and to gain better insights into ecosystem functions and processes (Cadotte, 2017; Díaz et al., 2004; Pérez‐Harguindeguy et al., 2013). Studying the variation in plant traits has become increasingly popular in ecology (Díaz et al., 2016; Kattge et al., 2011). For a large number of plant species and from a huge number of studies and sites, functional traits have been collated into large databases (Kattge et al., 2011; Kleyer et al., 2008; Kühn, Durka, & Klotz, 2004) but glaring taxonomic and geographical gaps remain (Jetz et al., 2016; Schrodt et al., 2015), especially in tropical ecosystems and remote regions (Schrodt et al., 2015). One main limitation to fill these gaps is that measuring functional traits in the field is often laborious or requires expensive equipment.

Leaf area is among the most important plant traits (Díaz et al., 2016; Pérez‐Harguindeguy et al., 2013; Violle et al., 2007; Wilson, Thompson, & Hodgson, 1999) and can be regarded as key trait relevant to other traits like the specific leaf area. Specific leaf area in turn is often used in growth form analyses (Evans & Poorter, 2001; Pérez‐Harguindeguy et al., 2013). It is also a key trait in the leaf economics spectrum (Wright et al., 2004), linked to differences in plant life strategies (Wilson et al., 1999), and correlates positively with photosynthetic rate, leaf nitrogen concentration, light interception, and relative growth rate and negatively with leaf longevity and carbon investment (Pérez‐Harguindeguy et al., 2013). Other important ecophysiological attributes of plants including leaf phosphorous capacity, dark respiration, chemical composition, and evapotranspiration are often expressed per leaf area (Garnier et al., 2017; Reich et al., 1999; Wright et al., 2004), emphasizing the importance of leaf area in plant ecology.

Measuring leaf area can be difficult under field conditions as standard protocols require a scanner, computer, and digital image processing by sophisticated and often expensive software to obtain accurate and reliable results (e.g., *Delta‐T Devices* (Cambridge, UK), *LI‐COR* (Lincoln, NE, USA), and *WinFOLIA* (Regent Instruments Canada Inc.)). This often restricts analyses of leaf area to laboratories with connection to electricity and computers (but see Pérez‐Harguindeguy et al. (2013) for low‐tech options for the measurement of leaf area).

Smartphones have a high potential for science (Welsh & France, 2012) as they are widespread, have strong computing power (Lane et al., 2010), and include a wide range of accurate tools like GPS, camera, and different types of sensors (e.g., acceleration sensors, gyroscopes, magnetic field sensors, light sensors, barometers, thermometers, and air humidity sensors). Smartphone applications using this set of sensors can be well suited to assist within fieldwork (Welsh & France, 2012), especially, as many applications are free of charge. Despite the many accurate sensors in smartphones, surprisingly few applications have been designed as tools for ecology and evolution (but see Teacher, Griffiths, Hodgson, & Inger, 2013) and are an underexploited resource. Also, the use of smartphones for plant functional ecology is highly undervalued. Only a few recent developments have been made to use smartphones for measuring plant traits like leaf area index (e.g., *PocketLA*I (Confalonieri, Francone, & Foi, 2014), *VitiCanopy* (De Bei et al., 2016)) and leaf area (*Petiole* (http://petioleapp.com/), *Easy Leaf Area* (Easlon & Bloom, 2014)).

Here, we present Leaf‐IT, a new smartphone application to measure leaf area as well as other trait‐related areas accurately under field conditions typical for remote locations.

Leaf‐IT uses a margin detection algorithm, that is, highly robust against unwanted shadows and impurities, which may interfere with area measurement. This makes Leaf‐IT fundamentally different to other area‐analyzing software and applications based on threshold‐based pixel count measurement (Easlon & Bloom, 2014). Leaf‐IT is specifically designed to measure the area under challenging field conditions, includes easy‐to‐use features for area measurement and data output, and can be used freely for ecological research and teaching. We tested the accuracy and precision of Leaf‐IT using real leaves as well as objects with standardized area and compared the results with the well‐established, commercial software WinFOLIA.

## 2 METHODS

### 2.1 Technical details of the application and margin detection

**Figure 1:**
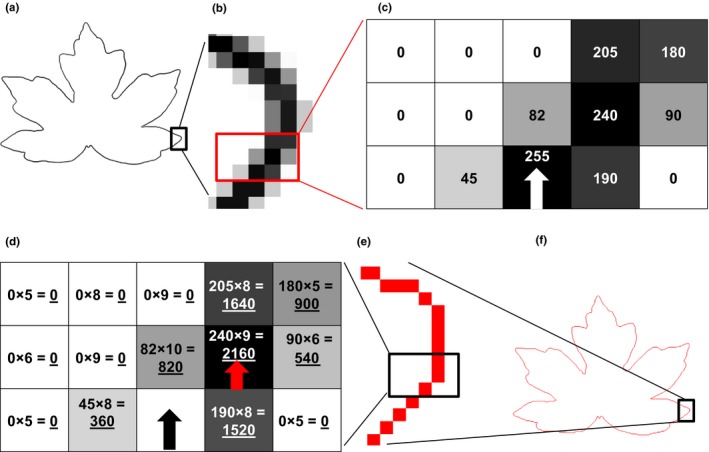
Details of image processing and pathway of the logical agent in Leaf‐IT. (a) Image of leaf after three steps of image processing and calculation of light gradients. Only the margin remains, shown as several‐pixel‐strong line (b). (c) The logical agent starts at the pixel with highest light gradient (white arrow) and evaluates all pixels in its viewing area (five times three pixels). Light gradient values (ranging from 0 to 255) of pixels, position of agent and its viewing direction (white arrow) are shown. (d) The agent multiplies the light gradient values (first factor) with values depending on the distance from the agent's position (second factor). Highest product (products are underlined) indicates the pixel where the agent moves next (pixel with red arrow). After each step, the agent starts again with the evaluation of its viewing area. The path of the agent is indicated as one‐pixel‐strong red line (e) until it has circled the whole margin of the leaf (f) and reaches its starting point again

Leaf‐IT runs on smartphones with Android 4 (or higher) operating systems and does not require connection to the Internet or databases. Images of leaves or other objects are taken by the internal smartphone camera. After image acquisition, Leaf‐IT uses digital image processing for area measurement and proceeds in three steps: (1) margin detection of the leaf or any desired object that has clearly defined margins, (2) pixel count, and (3) comparison with a reference object with a known area. For best results, the leaf should be placed on a background with a high contrast to the leaf. A white background works best for darker leaves. For lighter objects such as flower petals, a black background might be more suitable. After image acquisition, Leaf‐IT conducts three steps of image processing: (1) converting the image to grayscales; (2) highlighting the margins by increasing the contrast, blurring weak margins, and enhancing strong margins; and (3) calculating the light gradients and displaying the light gradients (Figure 1c), so that the image only retains the margins (Figure 1a, b). Light gradients are calculated by comparing the contrast between neighboring pixels and by assigning values between 0 and 255 to each pixel. Neighboring pixels with high contrast get high values (e.g., from white pixel to black pixel: value of 255) and neighboring pixels with low contrast (e.g., light gray pixel to gray pixel: value of 50; and white pixel to white pixel: value of 0) get low values. Light values are later displayed as pixels ranging from white to black, whereas pixels with low light values are displayed brighter (value of 0 equals white), and pixels with high values are displayed darker (value of 255 equals black). This procedure reduces the effects of distortions from, for example, unwanted shadows or lines on a background paper that become weaker or even vanish and interfere less with the margin detection of the leaf.

During calculation of the light gradients, the pixel with the highest gradient in the image, which is normally part of the leaf margin, is stored. A logical agent (Wooldridge & Jennings, 1995), specially designed for margin detection, is placed on the pixel with the highest light gradient and traces the margin step by step by drawing a line which is one‐pixel strong until it reaches its starting point again. The agent is based on the concept of a robot following a line (Barraquand, Langlois, & Latombe, 1992). During each step along the margin, the agent conducts four tasks (according to Russell & Norvig, 2016). First, the agent creates a viewing area of three times five pixels, where the agent occupies one pixel in the center of a five‐pixel‐long margin (Figure 1b). The direction from the pixel occupied by the agent toward the center of the viewing area is the viewing direction (Figure 1c, d). In the second step, the agent calculates weighted light values for each pixel in its viewing area. The values for each pixel of the light gradients are multiplied with a value depending on the location of the pixel within the viewing area (Figure 1d). Pixels located closer to the position of the agent and the viewing direction get the highest multiplier (based on the *inverse square law*; Figure 1d). Thus, pixels directly in front of the agent and in line with the viewing direction are considered more likely to be part of the leaf margin and get higher multipliers (Figure 1d). In the third step, the agent moves to the position of the pixel with the highest weighted light level (Figure 1d). In the fourth step, the agent verifies if it moved at all (in case its former path led to a dead end) and if it reached the starting position again. Each time the agent moves, it indicates the covered way as a one‐pixel‐strong red line (Figure 1e, f). The user can view the red line encircling the object for verification whether the agent encircled the leaf correctly (Figure 1f).

Defined rules are provided for the agent (following Russell & Norvig, 2016) for the evaluation of its last actions and to undo its last moves in case of errors. The rules provide guidelines for the agent how to proceed if it reaches the margin of the images or if it ran into a dead end (in this case, the agent goes back one step and proceeds to the pixel with the second highest weighted light value). The agent also contains exit commands to avoid endless searches and loops in pathfinding. In this case, an error message appears for the user, and area measurement stops.

### 2.2 Area measurement

**Figure 2:**
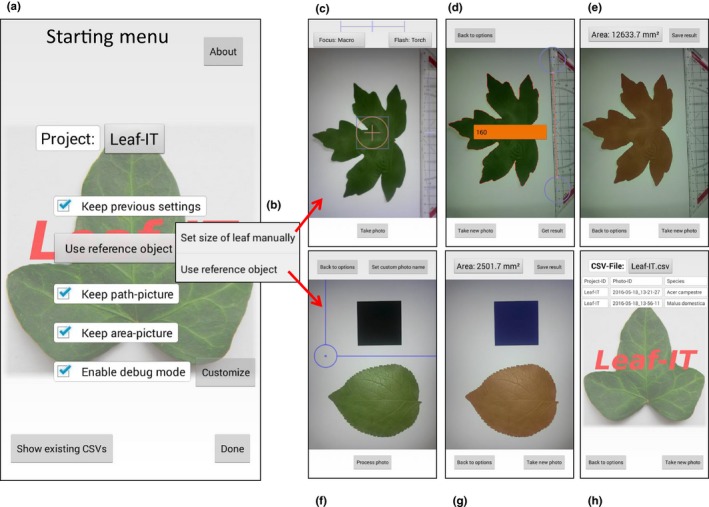
Starting menus, methods, and options in Leaf‐IT. (a) Starting menu with all relevant options displayed. (b) Options to choose between the two main methods (*set size* and *reference object*) for measurement leaf area and the nondestructive method. (c), (d), and (e) the different steps during the set size method, and (f) and (g) during the *reference object* method. (h) The output of Leaf‐IT can be exported as .csv‐file

After finishing the leaf margin detection, the area is measured. All pixels encircled by the one‐pixel‐strong red line are counted and compared with the number of pixel of a reference object of a known length or area. Two different methods are available in Leaf‐IT for setting a reference object. The first method (in Leaf‐IT: S*et size of leaf manually*; from now *set size*) allows the user to place an object of a known length (e.g., a ruler or any other defined object; compare Figure 2c) next to the leaf. By manually drawing a rectangle around the reference object, it is spared from image processing to not interfere with the margin detection. After margin detection, the user can adjust a digital ruler (which starts automatically; compare with Figure 2d) to the reference object and enter the length in mm. Next, the area of one pixel is calculated by counting the number of pixels of the digital ruler and set against the measured length. This allows the measurement of leaf area by comparing the number of pixels from the digital ruler and the leaf. The second method (in Leaf‐IT: *Use reference object*; from now *reference object*) allows the user to place an object with a known area (e.g., a coin or a printed rectangle; compare with Figure 2f) next to the leaf. Both reference object and leaf are processed separately (again by placing a digital rectangle around the reference object). After the image is processed, the user enters the area of the reference object. Leaf‐IT then compares the number of pixels of the reference object and the leaf and measures the area in cm^2^ as described above.

### 2.3 Tools, options, and data output

Leaf‐IT offers intuitive tools for data management, export, and image acquisition. All options can be selected and viewed in the Start menu (Figure 2a). The *Project* menu allows the user to create own projects. A project can be, for instance, a measurement series of a certain plant individual or species, a field site, or a sampling day. Each project can be exported as .csv‐file (Figure 2h). All area measurements within a project are saved in the same .csv‐file where also species names and image IDs can be edited or deleted (Figure 2h). The *set reference* menu contains the two methods how to define the reference object as described above (Figure 2b). Here, the user can select between *set size* (Figure 2c–e) and *reference object* (Figure 2f, g). After choosing the appropriate settings, Leaf‐IT opens the camera mode (Figure 2c). When the image mode is displayed, a level appears. Provided that the photographed object is in level, optimized setup for highest accuracy can thus be created (90° angle from camera lens to object; Figure 1c). After the image has been taken, the user defines the area where the reference object is located and proceeds to the image analysis as described above (Figure 2d, f). The detected margin is displayed in red with the image in the background (Figure 2d), allowing the user to evaluate the accuracy of the margin detection procedure before proceeding to area measurement. Here, the user defines length (method: *set size*) or area (method: *reference object*) of the reference object on the smartphone display (Figure 2d). The measured area of the leaf (Figure 2e, g) can be saved as a .csv‐file. The file also automatically includes the date and time of the area measurement and the image ID. All images as well as area and path images measured by Leaf‐IT (when requested in the *customize* option; Figure 2a) can be saved as .png in the Leaf‐IT folder or project subfolder on the smartphone where also the .csv‐file is saved.

### 2.4 Assessing accuracy and precision

Precision and accuracy are two important metrics for validating new measurement methods (Westgard, Carey, & Wold, 1974). Precision describes the random analytic error (distribution of the individual measurements around a mean value), while accuracy describes the systematic analytic error (difference between the mean of the measured values and the *true* value) (Westgard et al., 1974). We estimated both precision and accuracy of Leaf‐IT using standardized objects with known area. This allowed us to assess how accurate and precise Leaf‐IT reproduced the area and to compare measured and true leaf area.

For testing the accuracy of the *set size* method, we designed 22 shapes with different shapes and sizes (shapes are shown in Figure S1): eight different shapes with 1 cm^2^ and 10 cm^2^, respectively, and six different shapes with 100 cm^2^. Different shapes and areas were created in black color on white background with the software Microsoft PowerPoint Version 10 and printed out using a high‐resolution printer (Xerox Color 550, 2.400 dpi × 2.400 dpi) on 160 g/m^2^ paper. Precision and accuracy of the *reference object* method were measured on the same 22 objects as for the *set size* method. We only added a square of the same area next to the other object as reference area.

Subsequently, we compared the area match of real leaves of different sizes and morphologies between Leaf‐IT (*reference object* method) and the computer software WinFOLIA (Version: 2016b Pro; Regent Instruments Canada Inc., 2016). WinFOLIA is an established standard software for leaf area measurements.

### 2.5 Precision of Leaf‐IT

We measured the precision of Leaf‐IT using the *reference object* method (described above). Therefore, we took an image of the same object (a square) of the area classes of 1, 10, and 100 cm^2^ under optimized conditions (leveled smartphone with 90° angle between object and camera lens) ten times, respectively. Measured area was standardized for better comparison with the three area classes by dividing the measured area by ten for 10 cm^2^ and by 100 for 100 cm^2^. Thus, the true mean always equaled one. We calculated the precision for the three area classes (1, 10, and 100 cm^2^) separately. We indicated the precision (in %) by calculating the range between the lower and the upper confidence intervals (CI; upper CI minus lower CI).

### 2.6 Accuracy of Leaf‐IT

To test the accuracy of Leaf‐IT, we used the methods *set size* and *reference object* separately under optimized conditions (leveled smartphone, object in 90° angle from the lens) and handheld to simulate field conditions (four runs in total). All standardized objects were photographed and analyzed by Leaf‐IT (*n* = 22). Area values from each run were divided by 100 for 1 cm^2^, by 1,000 for 10 cm^2^, and by 10,000 for 100 cm^2^ for analyzing the three area classes together. We provided the accuracy (in %) by subtracting the calculated mean by the true mean (always one).

### 2.7 Comparison between Leaf‐IT and WinFOLIA

To test Leaf‐IT on real leaves, we compared the area measurements of Leaf‐IT with WinFOLIA. Therefore, we photographed 25 leaves of different size (from 1.88 to 115 cm^2^) and shape of 18 European plant species (species list and area values are provided in Table S1). The same photographs taken and analyzed by Leaf‐IT were also analyzed by WinFOLIA for direct comparison.

### 2.8 Statistical analyses

For testing the accuracy of Leaf‐IT, we compared the mean of the true area values of standardized objects with the area measured by Leaf‐IT. We calculated the differences (in %) and 95% CI of the area measured by Leaf‐IT toward the true area for all measurements of the same run, respectively (methods *set size*,* reference object*, and both methods combined under optimized conditions and handheld). For the precision, we calculated the mean and the 95% CI of ten measurements repeated on the same standardized object with the area of 1, 10, and 100 cm^2^, respectively. We used the Altman–Bland method (Altman & Bland, 1983; Bland & Altman, 1986) to compare area measurements of Leaf‐IT and WinFOLIA. This allowed us to investigate the relationship between the measurement error and the true value. However, as the true value was unknown, the mean of both measurements was the best estimate of the true value provided (Bland & Altman, 1986). We calculated the mean difference between both methods by subtracting the mean of the WinFOLIA measurements by the mean of the Leaf‐IT measurements. The mean difference indicated the bias of Leaf‐IT compared with WinFOLIA. The critical difference (in cm^2^) between both methods is expressed as the difference from the mean (of both methods) to the upper or lower 95% CI. All statistical analyses were performed in the statistical software *R* (version 3.3.1, R Core Team, 2017).

## 3 RESULTS

### 3.1 Precision of Leaf‐IT

**Figure 3:**
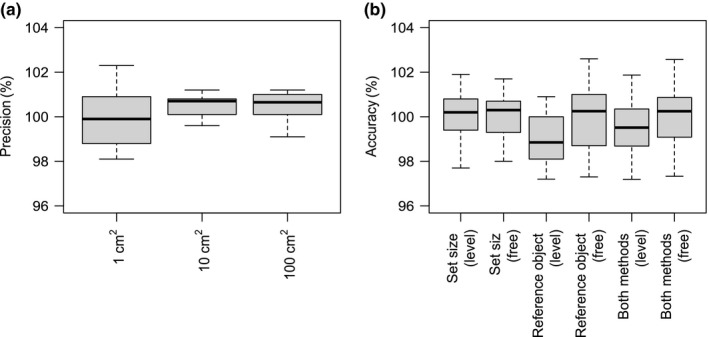
Precision and accuracy of Leaf‐IT. (a) Precision for reference objects of three area classes. The same area was measured ten times per class. (b) Accuracy of two methods IT (*set size* and *reference object*) for area measurement under optimized condition (level) and field conditions (free). Twenty‐two objects with known area were measured. In all cases, the true area equals 100%

For the smallest object size (1 cm^2^), the mean leaf area as measured by Leaf‐IT was exactly 1 (rounded by three decimal figures) showing that true and Leaf‐IT‐measured area values were virtually identical. The 95% CI was between 0.990 and 1.009 (*n* = 10) resulting in a precision of 98.1%. For the intermediate area class (10 cm^2^), the mean calculated from Leaf‐IT was 1.005, which differed from the true value by 0.5%. The 95% CI ranged from 1.001 to 1.009 (*n* = 10) with a precision of 99.2%. The largest area class (100 cm^2^) revealed a mean of 1.004, that is, 0.4% higher than the true value, and 95% CI ranged from 0.999 to 1.009 (*n* = 10) giving a precision of 99% (Figure 3a). All area measurements are provided in Table S2.

### 3.2 Accuracy of Leaf‐IT

We measured the accuracy of both Leaf‐IT methods (*reference object* and *set size*) under optimized conditions and under simulated field conditions. Using the *set size* method under optimized conditions, the mean was 1 (true mean also 1) with the 95% CI ranging from 0.996 to 1.005 (*n* = 22). Taking images under simulated field conditions, the calculated mean was 1.001, which gives a deviation of 0.1% from the true mean for the *set size* method. The 95% CI was between 0.997 and 1.005 (*n* = 22). Under optimized conditions, the method *reference object* produced a mean of 0.990 which deviated 1% from the true value. The 95% CI ranged between 0.986 and 0.995 (*n* = 22). The mean of simulating field conditions of the *reference object* method was 0.999 (0.1% of the true mean) with a 95% CI of 0.993 to 1.006 (*n* = 22; Figure 3b). All area measurements for the accuracy measurements are given in Table S3.

### 3.3 Leaf‐IT compared with WinFOLIA

**Figure 4:**
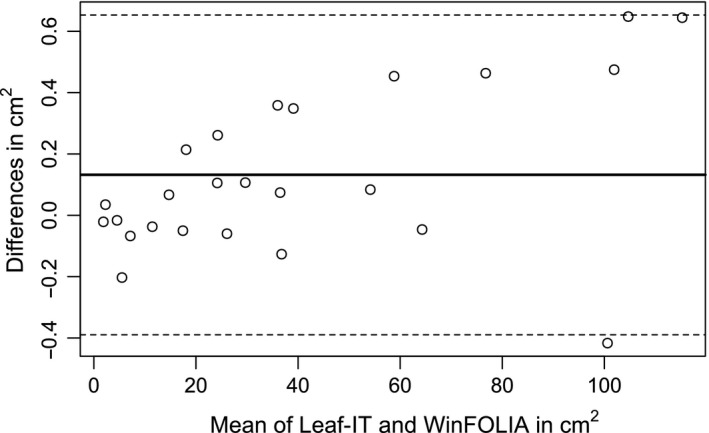
Bland–Altman plot showing the mean difference in leaf area measurements between Leaf‐IT and the commercial software WinFOLIA. Twenty‐five leaves of different sizes and shapes were measured by Leaf‐IT and WinFOLIA. The mean of area values for each leaf measured by WinFOLIA and Leaf‐IT is shown on the *x*‐axis. The *y*‐axis indicates the difference of each measurement of Leaf‐IT compared with WinFOLIA. Mean difference of all 25 measurements (solid line; 0.132 cm^2^) between both methods and 95% confidence intervals (dashed lines; 0.653 and −0.389 cm^2^) are shown

Area measured with Leaf‐IT was on average 0.1% (0.132 cm^2^) higher than that of WinFOLIA. The 95% CI ranged between −0.389 cm^2^ and +0.653 cm^2^ with a critical difference (half the difference from lower to upper CI) of 0.521 cm^2^. However, the highest mean difference was recorded for area values above 100 cm^2^. Smaller area values did not show larger difference than −0.203 cm^2^ and +0.463 cm^2^. The highest difference between two measured values was −3.6% and +1.5%. The mean difference between Leaf‐IT and WinFOLIA was +0.1% (Figure 4). Area measurements for different plant species estimated by Leaf‐IT and WinFOLIA are provided in Table S1.

## 4 DISCUSSION

Leaf‐IT is a new, easy‐to‐use, and free of charge application licensed under creative commons (license: CC BY‐NC‐SA 4.0) that produces sufficiently accurate and precise area measurements. Due to its intuitive graphical user interface and high portability, Leaf‐IT is useful for a wide range of applications in ecological research and teaching.

The logical agent and the option to choose between two different methods for area measurements make Leaf‐IT fundamentally different to other software programs that evaluate each pixel individually (e.g., WinFOLIA, Easy Leaf Area; Easlon & Bloom, 2014) or need elaborate image calibration (e.g., Petiole). Instead, Leaf‐IT encircles the leaf and rates each pixel equally within the enclosed area making Leaf‐IT more robust against shadows and other artifacts on the background. At the same time, Leaf‐IT currently has limitations in assessing leaf area of species with complex leaf morphologies (e.g., pinnate and fern leaves) and damaged leaves.

### 4.1 The *set size* method

The method *set size* yielded highly accurate results with a mean accuracy of less than 0.5%. The accuracy did not decrease when taking the image by handholding the smartphone, which conforms to challenging condition during fieldwork. Accuracy mainly depended on the accurate measurement of the reference object and the user skills to set the length on the smartphone display perfectly. Here, we recommend training before proceeding to real leaves by using a ruler as reference and a known area as object. The *set size* method, however, is more time‐consuming (about 40 seconds for a trained user from taking the image to obtaining the result) then the method *reference object* (about 30 seconds). Four separate manual steps are involved: (1) taking the image, (2) defining the patch where the reference object is located in the image, (3) measuring a distance on the reference object (can be simplified by using a ruler as reference), and (4) setting the length of the measured distance on the smartphone screen.

### 4.2 The *reference object* method

The method *reference object* by Leaf‐IT is also highly accurate (<1.5% deviation) and precise (2% deviation) under both optimized and field conditions. Based on our experience, highest accuracy can be achieved when camera lens and object are in perpendicular direction to each other. Furthermore, it should be avoided to fill out the whole image range provided by the camera with the reference object and the leaf. The closer the margins of the images lie to the object, the higher the image distortion becomes and increases the inaccuracy of the depicted objects. Different camera lenses and image sensors produced similar results in area measurements. We achieved reliable results by leaving blank about one‐third from the image margins toward the center. The method is, compared to the *set size* method, faster and more users friendly. Three manual steps are involved from taking the image to the results: (1) taking the image, (2) defining the patch where the reference object is located in the image, and (3) typing in the area of the reference object. For easy use, we recommend to use a printout (white paper) with a black square with known area in one corner (e.g., side length of 5 × 5 cm) serving as a reference object. The leaf can then be placed next to the reference object and both photographed together. The reference object and the unknown object should be roughly of the same size. During tests of the application in the field, it proved successful to have printouts prepared with reference objects ranging in area from the smallest to the highest leaf area expected.

### 4.3 Leaf‐IT compared with WinFOLIA

Leaf area measured in Leaf‐IT and WinFOLIA yielded similar results. The maximum difference between both methods was −3.6% and +1.5%, and the difference between the mean from Leaf‐IT and WinFOLIA was 0.132 cm^2^. These low values indicate that no method is biased toward the other and that both methods measure area equally well (Bland & Altman, 2003). For smaller leaves (<100 cm^2^), the difference of the means was <0.5 cm^2^ and decreased with leaf size. That means that the critical difference (0.521 cm^2^) was only recorded for the biggest leaves. When comparing area measurements of both methods for each leaf individually, the difference was always <4%. In 19 of 25 leaves, it was even smaller than 1%. When images showed shadows or the background had impurities, Leaf‐IT measured leaf area more reliable than WinFOLIA, which often had problems to distinguish between artifacts and real leaves. We choose for comparison only leaves which had simple margin morphologies and were undamaged. Here, Leaf‐IT detected the margin very accurately. However, when using damaged leaves or complex margin morphologies (e.g., ferns), Leaf‐IT may not have detected the margin correctly or detected at all.

### 4.4 Strengths and limitations of Leaf‐IT

All features in Leaf‐IT are specially designed for scientific use. Export of data comes as .csv‐file which can be imported to most common software programs for further data analyses. The option to choose between two methods (*set size* and *reference object*) allows the user to assess leaf area with minimal effort and preparation. Its high accuracy and precision are similar to those of other well‐established software (e.g., WinFOLIA). Different smartphone types can produce reliable results as we did not find great dissimilarities in area measurements related to lenses and image sensors. Its major limitation, however, is the margin detection of complicated leaf morphologies. Serrated, compound, pinnate, and strongly pilose or lobed leaves often cause problems for Leaf‐IT. This is, for instance, the case for some herbs (like many species from the families Apiaceae, Geraniaceae, Ranunculaceae, and Fabaceae) as well as ferns and plant species with similar leaf morphologies. Also, holes (as in *Monstera deliciosa* Liebm.) and herbivore damage within leaves cannot be detected by Leaf‐IT and are included in the overall leaf area.

## 5 CONCLUSION

In summary, Leaf‐IT is easy to use and applicable on all smartphones operating on Android 4 or higher. Android is the most widely used operating systems found on the widest range of smartphones (Teacher et al., 2013) increasing the portability and use of Leaf‐IT. Besides leaf area, all objects can be measured given a high light contrast of object and background. However, its main limitation is the area measurement of complex leaf morphologies. Here, further effort is needed to improve the performance with complex leaf morphologies. Collaborative testing of interested users could improve Leaf‐IT and provide more detailed suggestions and recommendation about strength and limitations of the application as well as to compile guidelines for future improvements on Leaf‐IT. We hope that Leaf‐IT motivates ecologists to use free smartphone applications designed for assessing functional traits in particular and for ecological data acquisition in general.

Leaf‐IT can be downloaded from: https://play.google.com/store/apps/details?id=de.yahoo.gisopillar.leafit

## AUTHOR CONTRIBUTIONS

JS and GP conceived the ideas and designed methodology. GP programmed the application. JS collected the data and analyzed the data. JS and HK led the writing of the manuscript. All authors contributed critically to the drafts and gave final approval for publication.

## CONFLICT OF INTEREST

The authors declare that they have no conflict of interests.
