# Supplementary material for: Leaf‐IT: An Android application for measuring leaf area
Source: Ecol Evol. 2017 Oct 18;7(22):9731–8. doi: 10.1002/ece3.3485 (PMC5696424; doi:10.1002/ece3.3485)
Supplement: Supplementary file 1 [file ECE3-7-9731-s001.docx]

# Supplementary Information

#### **S1:** Eight different shapes (black) and reference object (grey) used for testing accuracy and precision of Leaf-IT. Different shapes were created with the software Microsoft PowerPoint Version 10 and printed out using a high-resolution printer (Xerox Colour 550, 2.400 dpi x 2.400 dpi) on 160 g/m^2^ paper. Area of shapes and reference objects shown here is 1 cm^2^. For testing accuracy and precision for different area classes, the same shapes were also printed in 10 cm^2^ 100 cm^2^ respectively.

#### **S1:** Species list of 25 leaves with measured area by Leaf-IT and WinFOLIA. The difference indicates the area value measured by WinFOLIA minus the value measured by Leaf-IT. The accordance shows the similarity in % of the area values measured by both methods (area value of WinFOLIA equals 100%). All numbers are rounded by two decimal figures. Nomenclature follows: The Plant List, Version 1.1; accessed: August 2016.

| Species | Leaf-IT (cm^2^) | WinFOLIA (cm^2^) | Difference (cm^2^) | Accordance (%) | Mean Leaf-IT & WinFOLIA (cm^2^) |
| --- | --- | --- | --- | --- | --- |
| *Veronica hederifolia* | 1.88 | 1.86 | -0.02 | 99 | 1.87 |
| *Lonicera xylostemon* | 2.26 | 2.29 | 0.03 | 102 | 2.27 |
| *Symphoricarpus albus* | 4.55 | 4.53 | -0.02 | 100 | 4.54 |
| *Populus nigra* | 5.61 | 5.41 | -0.20 | 96 | 5.51 |
| *Lonicera xylostemon* | 7.18 | 7.11 | -0.07 | 99 | 7.15 |
| *Crataegus monogyna* | 11.47 | 11.43 | -0.04 | 100 | 11.45 |
| *Populus nigra* | 14.68 | 14.75 | 0.07 | 100 | 14.71 |
| *Sambucus nigra*^L^ | 17.47 | 17.42 | -0.05 | 100 | 17.44 |
| *Acer campestre* | 17.96 | 18.18 | 0.21 | 101 | 18.07 |
| *Prunus padus* | 24.12 | 24.23 | 0.11 | 100 | 24.18 |
| *Hedera helix* | 24.13 | 24.39 | 0.26 | 101 | 24.26 |
| *Malus domesticus* | 26.11 | 26.05 | -0.06 | 100 | 26.08 |
| *Plantago lanceolata* | 29.62 | 29.73 | 0.11 | 100 | 29.67 |
| *Tilia cordata* | 35.83 | 36.19 | 0.36 | 101 | 36.01 |
| *Malus domesticus* | 36.46 | 36.53 | 0.07 | 100 | 36.50 |
| *Prunus padus* | 36.85 | 36.72 | -0.13 | 100 | 36.78 |
| *Ribes rubrum* | 38.92 | 39.26 | 0.35 | 101 | 39.09 |
| *Allearia petiolata* | 54.12 | 54.20 | 0.08 | 100 | 54.16 |
| *Acer campestre* | 58.59 | 59.04 | 0.45 | 101 | 58.82 |
| *Carpinus betulus* | 64.33 | 64.28 | -0.05 | 100 | 64.31 |
| *Syringa vulgaris* | 76.53 | 76.99 | 0.46 | 101 | 76.76 |
| *Hedera helix* | 100.83 | 100.41 | -0.42 | 100 | 100.62 |
| *Acer campestre* | 101.68 | 102.16 | 0.47 | 100 | 101.92 |
| *Aesculus hippocastanum^L^* | 104.35 | 105.00 | 0.65 | 101 | 104.68 |
| *Arctium sp.* | 114.93 | 115.57 | 0.64 | 101 | 115.25 |

**^L - leaflet^**

#### **S2:** Precision of Leaf-IT. For each area class the same object was measured 10 times respectively (with *reference object* method in Leaf-IT). The values estimated by Leaf-IT are shown with mean and confidence intervals (CI). All numbers are rounded by two decimal figures.

|  | Area class | | |
| --- | --- | --- | --- |
| run | 1 cm^2^ | 10 cm^2^ | 100 cm^2^ |
| 1 | 0.99 | 10.07 | 100.58 |
| 2 | 1.01 | 10.08 | 100.37 |
| 3 | 1.00 | 10.12 | 100.07 |
| 4 | 1.02 | 10.10 | 100.78 |
| 5 | 1.00 | 9.96 | 99.26 |
| 6 | 0.99 | 10.02 | 100.98 |
| 7 | 0.99 | 10.01 | 101.23 |
| 8 | 1.00 | 10.07 | 100.70 |
| 9 | 1.02 | 9.97 | 99.10 |
| 10 | 0.98 | 10.07 | 101.00 |
| mean | 1.00 | 10.05 | 100.41 |
| CI lower | 0.99 | 10.01 | 99.90 |
| CI upper | 1.01 | 10.09 | 100.90 |

#### **S3:** Estimated area by Leaf-IT on 22 standardised object. The *true area* indicates the standardised area of the objects. *Form*, *length* and *width* show the properties of the objects. All objects were measured with the *set size* and *reference object* method in Leaf-IT under optimized conditions (level) and under field conditions (free). All numbers are rounded by two decimal figures.

| true area (cm^2^) | form | length (cm) | width (cm) | set size (level)  (cm^2^) | set size (free) (cm^2^) | reference object (level) (cm^2^) | reference object (free) (cm^2^) |
| --- | --- | --- | --- | --- | --- | --- | --- |
| 1 | square | 1.00 | 1.00 | 1.01 | 1.01 | 0.99 | 1.00 |
| 1 | rectangle | 0.50 | 2.00 | 1.01 | 1.00 | 0.99 | 0.99 |
| 1 | rectangle | 0.20 | 5.00 | 0.98 | 0.98 | 0.99 | 0.98 |
| 1 | circle | 1.13 | 1.13 | 1.01 | 0.99 | 1.00 | 1.01 |
| 1 | ellipse | 0.40 | 3.18 | 1.01 | 1.00 | 1.00 | 1.02 |
| 1 | triangle | 1.00 | 2.00 | 0.98 | 1.02 | 0.98 | 1.00 |
| 1 | triangle | 2.00 | 1.00 | 1.00 | 1.01 | 1.01 | 0.98 |
| 1 | triangle | 0.50 | 4.00 | 1.00 | 1.00 | 1.01 | 1.01 |
| 10 | square | 3.16 | 3.16 | 10.13 | 9.97 | 9.87 | 9.87 |
| 10 | rectangle | 1.58 | 6.32 | 9.92 | 9.93 | 9.79 | 10.03 |
| 10 | rectangle | 0.63 | 15.81 | 9.92 | 9.90 | 9.72 | 9.83 |
| 10 | circle | 3.57 | 3.57 | 10.19 | 10.16 | 9.79 | 10.18 |
| 10 | ellipse | 1.26 | 10.07 | 9.94 | 9.90 | 10.00 | 10.05 |
| 10 | triangle | 3.16 | 6.32 | 9.93 | 10.07 | 9.88 | 10.03 |
| 10 | triangle | 6.32 | 3.16 | 9.95 | 10.09 | 9.98 | 10.13 |
| 10 | triangle | 1.58 | 12.65 | 9.99 | 10.07 | 9.83 | 10.26 |
| 100 | square | 10.00 | 10.00 | 100.22 | 100.35 | 100.25 | 100.22 |
| 100 | rectangle | 5.00 | 20.00 | 100.20 | 100.49 | 99.28 | 101.05 |
| 100 | circle | 11.28 | 11.28 | 100.79 | 100.82 | 97.77 | 97.33 |
| 100 | ellipse | 4.00 | 31.83 | 99.53 | 99.08 | 100.17 | 100.54 |
| 100 | triangle | 10.00 | 20.00 | 100.84 | 99.52 | 97.89 | 99.09 |
| 100 | triangle | 20.00 | 10.00 | 99.36 | 101.03 | 98.13 | 97.39 |
